# Trastuzumab treatment improves brain metastasis outcomes through control and durable prolongation of systemic extracranial disease in HER2-overexpressing breast cancer patients

**DOI:** 10.1038/sj.bjc.6604941

**Published:** 2009-02-24

**Authors:** Y H Park, M J Park, S H Ji, S Y Yi, D H Lim, D H Nam, J-I Lee, W Park, D H Choi, S J Huh, J S Ahn, W K Kang, K Park, Y-H Im

**Affiliations:** 1 Division of Hematology/Oncology, Department of Medicine, Samsung Medical Center, Sungkyunkwan University School of Medicine, Seoul, Korea; 2 Department of Neurosurgery, Samsung Medical Center, Sungkyunkwan University School of Medicine, Seoul, Korea; 3 Department of Radiation Oncology, Samsung Medical Center, Sungkyunkwan University School of Medicine, Seoul, Korea

**Keywords:** trastuzumab, brain metastasis, HER2-overexpressing breast cancer, extracranial disease control

## Abstract

In patients with human epidermal growth factor receptor-2 (HER2)-overexpressing breast cancer, treatment with trastuzumab has been shown to markedly improve the outcome. We investigated the role of trastuzumab on brain metastasis (BM) in HER2-positive breast cancer patients. From 1999 to 2006, 251 patients were treated with palliative chemotherapy for HER2-positive metastatic breast cancer at Samsung Medical Center. The medical records of these patients were analysed to study the effects of trastuzumab on BM prevalence and outcomes. Patients were grouped according to trastuzumab therapy: pre-T (no trastuzumab therapy) *vs* post-T (trastuzumab therapy). The development of BM between the two treatment groups was significantly different (37.8% for post-T *vs* 25.0% for pre-T, *P*=0.028). Patients who had received trastuzumab had longer times to BM compared with patients who were not treated with trastuzumab (median 15 months for post-T group *vs* 10 months for pre-T group, *P*=0.035). Time to death (TTD) from BM was significantly longer in the post-T group than in the pre-T group (median 14.9 *vs* 4.0 months, *P*=0.0005). Extracranial disease control at the time of BM, 12 months or more of progression-free survival of extracranial disease and treatment with lapatinib were independent prognostic factors for TTD from BM.

There has been a steady decline in the death rate from breast cancer worldwide since 1990. The development of new treatments, such as a human epidermal growth factor receptor-2 (HER2)-directed monoclonal antibody, has offered new hope to women with both early and advanced breast cancer whose tumours overexpress HER2. However, despite these clinical advances, as well as advances in our understanding of the related biology, breast cancer is still the main cause of cancer morbidity and mortality in women in most countries (Mollick and Carlson, 2004; Boyle and Ferlay, 2005; Smigal *et al*, 2006; Korea National Statistical Office, 2007). In HER2-overexpressing breast cancer, which is associated with a high recurrence rate and poor outcome (Slamon *et al*, 1987; Paik *et al*, 1990; Kallioniemi *et al*, 1991; Boss *et al*, 2003), trastuzumab-based therapies are well established and regarded as a standard therapy (Slamon *et al*, 2001; Pegram *et al*, 2004). Since the introduction of trastuzumab, different groups have reported a higher incidence of brain metastases (BMs) in their patients ranging from 28 to 43% (Bendell *et al*, 2003; Altaha *et al*, 2004; Clayton *et al*, 2004; Lin *et al*, 2004; Souglakos *et al*, 2006; Stemmler *et al*, 2006; Yau *et al*, 2006). The biological explanation for the high incidence of BM may be a greater affinity of HER2-overexpressing breast cancer for the brain (Ross *et al*, 2003; Gori *et al*, 2007; Lin and Winer, 2007). Additionally, it may be argued that trastuzumab-based therapy prolongs survival to such an extent that BM, which is known to be a late event in the course of metastatic cancer, becomes apparent. The higher incidence of BM could be interpreted as a result of the prolonged duration of the disease (Stemmler and Heinemann, 2008).

Central nervous system (CNS) metastases, including parenchymal and leptomeningeal metastases, are the most common causes of malignant disease in the brain. Historically, the lack of durability for CNS control was not a problem for most patients because BMs occurred late in the course of illness, and progression in non-CNS was the dominant cause of morbidity and mortality (Herrero *et al*, 2004; Bartsch *et al*, 2007). However, as systemic therapies improve, the control of CNS disease seems to become increasingly important for overall disease control.

The purpose of this retrospective study was to analyse how the addition of trastuzumab to conventional chemotherapies affects the clinical course of BM in patients with HER2-overexpressing metastatic breast cancer (MBC), in terms of its biological behaviour of HER2.

Secondly, we characterised the prevalence and predictors of CNS metastasis among patients with HER2-overexpressing MBC receiving trastuzumab-based therapy.

## Patients and methods

### Study design and population

We conducted a retrospective analysis of medical records from patients with histologically confirmed metastatic or recurrent breast cancer who received palliative chemotherapy at Samsung Medical Center from January 1999 to December 2006. All pathological specimens were reviewed by two experienced pathologists. HER2 status was evaluated using an antibody (CBII, Novocastra Laboratories, Newcastle, UK) and fluorescence *in situ* hybridisation (FISH). Grade 3 of the HER2 by immunohistochemical (IHC) staining was defined as a positive result for HER2, and amplification of the HER2 was confirmed by FISH if HER2 was rated 2+ by IHC. The pre-trastuzumab period (pre-T) was from 1999 to 2002. Most of the patients in this period did not receive trastuzumab because it was unavailable or not reimbursed by the Korean medical insurance system for clinical use. We used the patients in this period as the ‘historical control’. The post-trastuzumab period (post-T) was from 2003 to 2006. Trastuzumab was available and reimbursed by the insurance system in Korea for HER2-overexpressing breast cancer patients during this time period.

Treatment modalities for BMs, either single or combined modalities, included whole brain radiation therapy (WBRT), surgical resection, stereotactic radiosurgery (SRS), including gamma-knife surgery (GKS) and systemic treatments such as chemotherapy and endocrine therapy. GKS was repeated for symptomatic control of metastatic brain lesion(s) if possible, with regular brain MRI follow-ups. Our study protocol was approved by the institutional review board of Samsung Medical Center.

### Statistical analysis

The distant metastasis-free survival (DFS) was defined from the date of breast cancer diagnosis to the date of documentation of distant metastasis. The time to BM (TTBM) was defined from the date of distant metastasis to the date of BM. The time to death (TTD) from BM was defined from the date of BM to death or the last follow-up day. The overall survival (OS) was measured from the first day of treatment for MBC to the date of death or to the last follow-up day. The progression-free survival (PFS) of extracranial disease was defined from the first day of last chemotherapy regimen that had been administered prior to BM, to the date of progression of systemic disease irrespective of development or progression of CNS metastases. A switch in chemotherapy after development or progression of BM was allowed, and was not regarded as progression of extracranial disease. The brain PFS was defined from the last date of treatment for CNS metastasis to the date of progression of any CNS sites. Extracranial disease control was defined as systemic disease status, except in the CNS, with complete response (CR), partial response (PR) or stable disease (SD) at the time of BM. ‘Only BM’ was defined as absence of evidence of other distant metastasis except CNS. ‘First metastasis in brain’ was defined as the presence of brain involvement with or without systemic metastases when MBC was first documented. The OS, TTD, DFS, extracranial PFS and brain PFS were estimated by the Kaplan–Meier product limit method. The log-rank test was used to compare survival rates. A *P*-value <0.05 was considered significant. The differences in characteristics between pre-T and post-T were estimated by the *χ*^2^ test or Fisher's exact test. A binary logistic regression analysis was used for the multivariate analysis of each potential prognostic variable for development. A Cox proportional hazards regression model was used to assess the effect of each potential prognostic variable on TTD and OS. All potential prognostic variables were included in the model, and variables were removed from this model one at a time in a backward selection process using the likelihood ratio and a significance level of 0.05.

## Results

### Patient population

**Figure 1:**
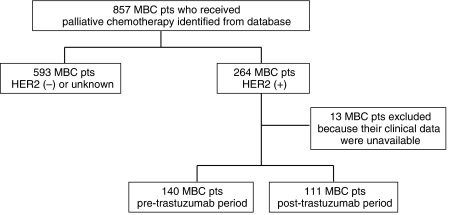
Patients cohort.

We identified 857 MBC patients who received palliative chemotherapy for the treatment of metastatic or recurrent breast cancer at Samsung Medical Center from 1999 to 2006. Two hundred sixty-four patients of the 857 patients were confirmed as HER2-overexpressing breast cancer patients. Among these patients, 140 patients were not treated with trastuzumab in pre-T period and 111 patients were treated with trastuzumab in post-T period. And remaining 13 patients were excluded from the analysis because of unavailability of their clinical data. Therefore, we included a total of 251 patients in this study (Figure 1).

### Development of brain metastasis

The incidence of BM among the patients in this study was 30.7% (77 out of 251). The incidence of BM in each treatment group was 25.0% (35 out of 140) in the pre-T group and 37.8% (42 out of 111) in the post-T group (*P*=0.028 by *χ*^2^ test).

### Patients' characteristics at the time of brain metastasis

All 251 patients' characteristics are summarised in Table 1. The median age of the entire population was 48 (range 25–77). Common metastatic sites were lung (57.0%), bone (50.8%), lymph nodes (37.9%), liver (41.7%) and brain (30.7%). There was no difference between the two groups in terms of metastatic organs, median age, stage and disease status. Grade III nuclear grades are more common in post-T group than pre-T group (40.2 *vs* 58.8%, *P*=0.014).

The patients' characteristics of 77 HER2-overexpressing MBC patients at the time of BM are summarised in Table 2. The number of patients who had three or more metastatic brain lesions was 28 (80.0%) in the pre-T group and 27 (64.3%) in the post-T group (*P*=0.129). Leptomeningeal seeding had developed in three pre-T patients (8.6%) and nine post-T patients (21.4%). There was no significant difference between pre-T and post-T patients in terms of hormonal receptor status, distribution of initial TNM staging, pathological grade or performance status (PS). However, disease control rate of systemic extracranial lesions (CR+PR+SD) at the time of BM was much higher in the post-T group (75.6%) than in the pre-T group (48.1%) (*P*=0.020). The ‘only BM’ was more common in pre-T patients (14.3%) than in post-T patients (2.4%) (*P*=0.046). In addition, the development of the ‘first metastasis in brain’ was also more common in the pre-T group (25.7%) than in the post-T group (9.5%) (*P*=0.058).

### Trastuzumab treatment

Trastuzumab was administered to 27 of the 42 patients with BM (64.3%) as a first-line treatment with cytotoxic chemotherapy. Of the remaining 15 patients (35.7%), 10 were treated with trastuzumab as a second-line therapy, and three were treated with trastuzumab as a third- or more line therapy. There were 12 patients (28.6%) who had started trastuzumab after development of BM. Two patients were treated with trastuzumab continuously before and after BM. The median duration of trastuzumab treatment was 6.4 (1.2–22.5) months. Lapatinib was also administered in 10 patients (23.8%) for salvage treatment after BM.

### Local and systemic treatment for brain metastasis

Craniotomy was performed in nine patients (11.7%) (8.6% in pre-T *vs* 14.3% in post-T, *P*=0.437). GKS was more common in the post-T group (59.5%) than in the pre-T group (28.6%) (*P*=0.007). WBRT was performed in 69 patients (89.6%) (91.4% in pre-T *vs* 81.1% in post-T, *P*=0.633). Systemic chemotherapy was delivered to 45 patients (58.4%) and the median number of the systemic chemotherapy regimens was 1 (range 0–6). The patients who were treated with 1 or more systemic chemotherapy regimens were much more common in the post-T group than in the pre-T group (57.1 *vs* 11.4%, *P*<0.0001). The median number of regimens delivered to patients was 0 (0–4) in pre-T patients and 2 (0–6) in post-T patients. The agents used in combination or as single systemic treatment regimens with or without trastuzumab after BM were anthracycline, taxane(s), gemcitabine, cisplatin, capecitabine and vinorelbine.

### Brain metastasis outcomes and logistic regression univariate analysis on TTD

**Figure 2:**
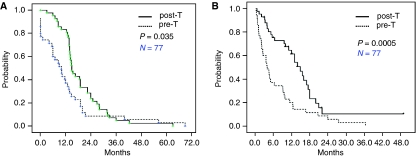
Brain metastasis outcomes of 77 HER2-overexpressing breast cancer patients with CNS metastasis. (**A**) Time to brain metastasis (TTBM) from metastasis; Solid (upper) line represents TTBM of 42 patients with CNS metastasis in the post-trastuzumab period. Median TTMB was 15.0 months (95% CI 12.5–17.5). Dotted (lower) line represents TTMB of 35 patients with CNS metastasis in the pre-trastuzumab period. Median TTMB was 10 months (95% CI 6.1–13.9) (*P*=0.035 by log-rank test). (**B**) Time to death (TTD) from brain metastasis; Solid (upper) line represents TTD of 42 patients with CNS metastasis in post-trastuzumab period. Median TTMB was 14.9 months (95% CI 11.6–18.2). Dotted (lower) line represents TTMB of 35 patients with CNS metastasis in pre-trastuzumab period. Median TTMB was 4.0 months (95% CI 2.1–5.9) (*P*=0.0005 by log-rank test).

**Figure 3:**
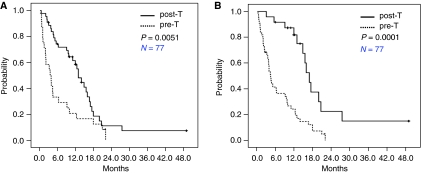
Kaplan–Meier survival curves of TTD according to extracranial systemic disease control at the time of brain metastasis and TTD according to the duration of extracranial systemic disease control, except for that in the CNS. (**A**) TTD according to systemic disease control at the time of brain metastasis; Solid (upper) line represents TTD of the patients with extracranial systemic disease control (CR or PR or SD) at the time of brain metastasis. Median TTD was 13.0 months (95% CI 10.6–15.4). Dotted (lower) line represents TTD of the patients without extracranial systemic disease control (PD) at the time of brain metastasis. Median TTD was 3.6 months (95% CI 1.4–5.8) (*P*=0.0051 by log-rank test). (**B**) TTD according to duration of systemic disease control, except for that in the CNS; Solid (upper) line represents TTD of the patients with 12 months or more PFS with systemic disease, not including CNS. Median TTD was 17.0 months (95% CI 14.8–19.2). Dotted (lower) line represents TTD of the patients with less than 12 months PFS of systemic disease, not including CNS. Median TTD was 4.5 month (95% CI 3.6–5.4) (*P*<0.0001 by log-rank test).

The OS and TTD from development of systemic metastasis were 26.4 months (95% CI 20.9–31.9) and 9.6 months (95% CI 4.6–14.6), respectively. The OS from development of systemic metastasis was longer in post-T patients (31.7 months; 95% CI 28.1–35.3) than in pre-T patients (16.7 months; 95% CI 10.3–23.1) (*P*=0.001). 1-year survival rate was significantly higher in post-T group than in pre-T group (60.0 *vs* 20.0%, *P*=0.001). TTBM was longer in the post-T group (15 months; 95% CI 12.5–17.5) than in the pre-T group (10 months; 95% CI 6.1–13.9) with statistical significance (*P*=0.035 by log-rank test) (Figure 2A). TTD from BM was also significantly longer in the post-T patients (14.9 months; 95% CI 11.6–18.2) than in the pre-T patients (4.0 months; 95% CI 2.1–5.9, *P*=0.0005) (Figure 2B). In a logistic regression model, lapatinib treatment (*P*=0.049), two or more systemic chemotherapy regimens after BM (*P*<0.0001), absence of ‘first metastasis in brain’ (*P*=0.045), extracranial disease control at the time of BM (*P*=0.0051) and 12 months or more extracranial PFS (*P*<0.0001) were identified as favourable prognostic factors for survival from BM (Figure 3A and B). 11.9% of the post-T patients and 37.1% of the pre-T patients died from the progression of extracranial systemic diseases, mainly lung and liver metastases (*P*=0.014 by Fisher's exact test) (Table 3).

### Cox-regression multivariate analysis of TTD

According to the results of univariate analysis, we performed Cox-regression multivariate analysis to identify independent factors for survival from BM. Table 4 summarises the Cox-regression model used in our study. Lapatinib treatment after BM (*P*=0.040, hazard ratio (HR) 5.069), two or more chemotherapy regimens after BM (*P*=0.001; HR 5.069), ‘first metastasis in brain’ (*P*=0.007, HR 10.390), extracranial disease control at the time of BM (*P*=0.009, HR 8.368) and extracranial PFS ⩾12 month (*P*<0.0001, HR 26.301) were identified as independent prognostic factors for favourable survival from BM. Trastuzumab treatment after BM also showed a high HR, though it did not reach statistical significance (*P*=0.086, HR 3.597).

## Discussion

Brain metastases generally occur late in the course of MBC and are associated with 1- and 2-year survival rates of only 20 and <2%, respectively, with most patients dying of systemic disease progression involving the lungs, liver and/or bone (DiStefano *et al*, 1979; Tsukada *et al*, 1983; Patanaphan *et al*, 1988; Engel *et al*, 2003). Several studies have examined the effect of HER2 status on the survival of patients after CNS metastases, and these studies describe shorter survival times from CNS metastases in patients with HER2-positive disease who never received trastuzumab compared with patients with HER2-negative disease (Kirsch *et al*, 2005; Tham *et al*, 2006). Some studies clearly show that even in the absence of trastuzumab, women with HER2-positive breast cancer have an increased propensity to develop CNS metastases compared with women with HER2-negative disease (Pestalozzi and Brignoli, 2000; Pestalozzi *et al*, 2006). Results from our study further illustrate that the incidence of CNS metastasis in patients with HER2-positive disease is significantly higher in patients treated with trastuzumab than in those not treated with trastuzumab (37.8 *vs* 25.0%, respectively, *P*=0.028). This finding can be explained by both the known aggressive nature and by the increased predilection to CNS metastases of HER2-positive tumours (Dawood *et al*, 2008). In addition, adding trastuzumab did not affect the development of BMs in HER2-overexpressing breast cancer, which is associated with the poor penetration of trastuzumab across the blood-brain barrier (BBB) and poor activity in BMs.

Considering that trastuzumab is known not to cross the BBB (Pestalozzi and Brignoli, 2000; Stemmler *et al*, 2007) and that patients treated with trastuzumab show higher development of BM than patients not treated with trastuzumab, introduction of trastuzumab might not overcome the biological aggressiveness of HER2-positive breast cancer, but may affect the clinical features of BM by improving systemic extracranial outcomes. Actually, a comparison of the demographic data between the pre-T and post-T groups in our series illustrated that ‘only BMs’ (14.3 *vs* 2.4%, respectively, *P*=0.046) is less common in the post-T group compared with the pre-T group (Table 2). Our understanding of these phenomena is that trastuzumab alone or in combination with chemotherapy seems to have an indirect, preventive or delaying effect on CNS involvement, which is caused by better systemic extracranial control with the addition of trastuzumab.

Most importantly, our study clearly shows that adding trastuzumab to conventional chemotherapy results in improved BMs outcomes in terms of a much longer TTBMs (15 months *vs* 10 months, *P*=0.035) and TTD (14.9 months *vs* 4.0 months, *P*=0.0005) that are independent of the trastuzumab line and duration (Figure 2). These results are consistent with recently published data from other studies (Bartsch *et al*, 2007; Dawood *et al*, 2008).

Taken together, our results reveal a higher incidence of BM in HER2-overexpressing breast cancer, but improved outcomes after introduction of trastuzumab. This finding strongly suggests that the level of control of extracranial disease, which is improved with trastuzumab, is a contributing factor to the timing of development of CNS metastases, and may be an important factor to be considered when building predictive models. Interestingly, 12 months or more extracranial PFS was identified as an independent prognostic factor with marked statistical significance (*P*<0.0001, HR 26.301) in addition to extracranial systemic disease control at the time of BM (*P*=0.009, HR 8.368) (Table 4). Only two patients received trastuzumab after development of BM. Therefore, after cessation of trastuzumab treatment there is evidence of a continuing or ‘carry-over’ effect that results in an increasing benefit after cessation of trastuzumab. This carry-over effect can create a more controllable extracranial condition with less tumour burden, which is important for additional systemic treatment as well as for active local treatment of BMs. As a result, survival from BM can be prolonged. This explanation is also supported by the finding that ‘two or more systemic chemotherapy regimens’ is an independent prognostic factor on TTD (*P*=0.001, HR 8.614). This finding confirms that an active systemic treatment has an important role and should be considered even in CNS metastases, because extracranial systemic control is a main contributing factor for predicting survival. It is commonly believed that most patients with BMs from advanced breast cancer do not die from cerebral progression, but rather from progression of systemic disease. This was apparently the major limiting factor in the survival of women before the trastuzumab period, and since the introduction of trastuzumab, the proportion of patients with controlled systemic disease dying from brain lesions has grown (Kirsch *et al* 2005; Lin and Winer, 2007). Apparently, death from progression of systemic disease was more common in the pre-T than in the post-T group (37.1 *vs* 11.9%, respectively, *P*=0.014) (Table 3). As shown in our study, it should be assumed that the impact on TTD stems from the control of extracranial systemic disease, rather than the control of brain lesions.

Further, it is important to be very cautious when stopping trastuzumab treatment or switching to other drugs, because it is difficult to identify cases of trastuzumab resistance.

Despite the emerging role of trastuzumab and the development of local treatments, such as SRS, including GKS, the overall outcome still needs to be improved. According to our earlier report, in which a prognostic model was suggested, TTD extended up to 49 months in cases of patients with no risk factor (good PS, HER2 negativity and additional systemic chemotherapy after BM) (Park *et al*, 2009). Considering TTD was merely 14.9 months even in post-T period, and lapatinib treatment was identified as an independent prognostic factor in Cox-regression model (*P*=0.040, HR 5.069) in this study (Table 4), new therapeutic strategies for BM in HER2-positive breast cancer are urgently needed and lapatinib may be a good therapeutic option in these cases.

The interpretations from this study have limitations. First of all, this is a retrospective single institutional study with a heterogeneous group of patients and treatments. However, this study population has some obstacles to develop a prospective randomised trial, such as relatively low prevalence, the urgent need for immediate supportive care and approval of the ethical problem to omit standard treatment. Taking allowance for these obstacles, this study has powerful value in providing guidelines for this particular clinical setting.

In conclusion, the administration of trastuzumab in the treatment of HER2-positive breast cancer results in prolonged TTBM and TTD from BM, even after development of BM. Extracranial systemic disease control at the time of BMs and durable prolongation of extracranial systemic disease are the main independent prognostic factors for predicting survival with BM.

## Figures and Tables

**Table 1:** Patient characteristics of 251 HER2-positive metastatic breast cancer patients

	**Pre-trastuzumab group (*n*=140)**	**Post-trastuzumab group (*n*=111)**	***P*-value (*χ*^2^ test)**
*Age*			
Median (year, range)	48 (25–77)	48 (25–71)	0.412
ER (+ ve)	63 (45.0%)	41 (36.9%)	0.198
PR (+ ve)	43 (30.9%)	34 (30.6%)	0.959
			
*Disease status*			
Recurrent	115 (82.1%)	83 (74.89%)	0.155
Initially metastatic	25 (17.9%)	28 (25.2%)	
Median DFS (log-rank test)	33.2 months	29.3 months	0.667
			
*Initial TNM stage* (n=239)			
I	16 (12.3%)	18 (16.5%)	0.386
II	42 (32.3%)	30 (27.5%)	
III	57 (43.8%)	42 (38.5%)	
IV	15 (11.5%)	19 (17.4%)	
			
Nuclear grade high (*n*=177)	39 (40.2%)	47 (58.8%)	0.014
Histological grade high (*n*=166)	37 (43.0%)	37 (46.3%)	0.676
Neoadjuvant chemotherapy (*n*=213)	26 (24.3%)	21 (19.8%)	0.430
Adjuvant chemotherapy (*n*=215)	110 (90.2%)	82 (88.2%)	0.640
Adjuvant radiation therapy (*n*=214)	69 (57.0%)	63 (67.7%)	0.110
			
*Metastatic sites*			
Lymph node (174)	50 (40.0%)	16 (32.7%)	0.529
Liver (*n*=240)	48 (37.2%)	52 (46.8%)	0.223
Lung (*n*=242)	72 (55.0%)	66 (59.5%)	0.481
Bone (*n*=242)	66 (50.4%)	57 (51.4%)	0.651
Brain (*n*=251)	35 (25.0%)	42 (37.8%)	0.028

DFS=distant metastasis-free survival; ER=oestrogen receptor; PR=progesterone receptor.

**Table 2:** Patient characteristics at the time of BM (*n*=77)

	**Pre-trastuzumab group (*n*=35)**	**Post-trastuzumab group (*n*=42)**	***P*-value (*χ*^2^ test)**
*Age*			
Median (year, range)	44 (25–66)	46 (34–70)	0.145
ER (+ ve) and/or PR (+ ve)	17 (48.6%)	15 (35.7%)	0.254
			
*Disease status*			
Recurrent	29 (82.8%)	39 (92.9%)	0.144
Initially metastatic	6 (17.2%)	3 (7.1%)	
			
*Initial TNM stage* (n=68)			
I	2 (6.3%)	3 (7.7%)	0.726
II	17 (53.1%)	17 (43.6%)	
III	13 (40.6%)	19 (48.7%)	
Nuclear grade high (*n*=64)	19 (70.4%)	25 (67.6%)	0.811
Adjuvant chemotherapy (*n*=68)	65 (79.3%)	64 (75.5%)	0.533
Adjuvant radiation therapy (*n*=68)	19 (59.4%)	25 (62.5%)	0.787
DFS ⩾24 months (*n*=68)	15 (46.9%)	21 (53.8%)	0.559
			
*Performance status*			
0–1	22 (62.9%)	30 (71.4%)	0.424
2⩽	13 (37.1%)	12 (28.6%)	
			
*Systemic disease status*			
CR/PR/SD	13 (48.1%)	31 (75.6%)	0.020
PD	14 (51.9%)	10 (24.4%)	
Number of BMs ⩾3	28 (80.0%)	27 (64.3%)	0.129
Leptomeningeal seeding	3 (8.6%)	9 (21.4%)	0.121
Only BM	5 (14.3%)	1 (2.4%)	0.046
First metastasis in brain	9 (25.7%)	4 (9.5%)	0.058

BM=brain metastasis; CR=complete response; DFS=distant metastasis-free survival; ER=oestrogen receptor; PD=progressive disease; PR=partial response; PR=progesterone receptor; SD=stable disease.

**Table 3:** Causes of death in 77 patients with BMs in HER2-positive breast cancer

	**Pre-trastuzumab group (*n*=35)**	**Post-trastuzumab group (*n*=42)**	***P*-value**
Brain	16 (45.7%)	25 (59.5%)	0.227 (*χ*^2^ test)
Extracranial systemic disease	13 (37.1%)	5 (11.9%)	0.014 (Fisher's exact test)
Lung	9 (25.7%)	3 (7.1%)	
Liver	3 (8.6%)	1 (2.4%)	
Heart	1 (2.8%)	1 (2.4%)	
Unknown	7 (17.1%)	13 (28.6%)	

BMs=brain metastases.

**Table 4:** Cox-regression multivariate analysis on time to death from BM (TTD)

			**95% CI**	
	**Significance (*P*-value)**	**Hazard ratio (HR)**	**Lower**	**Upper**
Lapatinib after BM	0.040	5.069	1.080	23.784
Number of post-BM chemotherapy regimens ⩾2	0.001	8.614	2.388	31.075
Post-BM trastuzumab	0.086	3.597	0.834	15.523
T-duration ⩾6 months	0.792	1.168	0.369	3.694
First metastasis in brain	0.007	10.390	1.919	56.256
Poor PS (⩾2)	0.464	1.473	0.522	4.153
Gamma-knife surgery	0.746	1.181	0.431	3.239
Extracranial systemic disease control at the time of BM	0.009	8.368	1.697	41.264
Systemic PFS ⩾12 months	<0.0001	26.301	4.871	142.004

BM=brain metastasis; CI=confidence interval; PFS=progression-free survival; PS=performance status; T-duration=duration of trastuzumab treatment.
